# Atypical Presentation of Gastric Cancer Approached Via Retrograde Single Balloon Enteroscopy

**DOI:** 10.7759/cureus.3249

**Published:** 2018-09-04

**Authors:** Hina Yousuf, Hafiz M. Kashif Saleem, Muhammad Arslan Arif Maan, Nadeem A Chaudhary

**Affiliations:** 1 Internal Medicine, Dow University of Health Sciences, Karachi, PAK; 2 Internal Medicine, Allama Iqbal Medical College, Lahore, PAK; 3 Clinical Oncology, Faisalabad Medical University Cancer Center, Faisalabad, PAK; 4 Gastroenterology, Regions Hospital, Saint Paul, MN, USA

**Keywords:** adenocarcinoma, gastric cancer, partial gastrectomy, single balloon enteroscopy, abdominal pain, case report

## Abstract

The paradigm for the diagnosis and management of gastric cancer is changing with advanced diagnostic and therapeutic interventions. Prior gastric surgery (20 years or more) is one of the risk factors for gastric cancer. Increased intragastric carcinogen formation is thought to contribute toward gastric cancer development in the remaining portion of the stomach. This case illustrates the importance of a thorough clinical and pathologic workup and highlights the advanced technique of single-balloon enteroscopy (SBE) and its role in managing patient’s health.

## Introduction

Gastric cancer is the fourth most common cancer and the second most common cause of cancer-related deaths globally [1]. Regular screening and early detection have decreased the incidence of stomach cancer in the Western world. The American Cancer Society estimates the risk of stomach cancer in the United States for 2018 as follows: about 26,240 cases of stomach cancer will be diagnosed (16,520 in men and 9,720 in women) and about 10,800 people will die from this cancer (6,510 men and 4,290 women) [2-3]. Different gastric malignancies with respect to their incidences are adenocarcinoma (90% to 95%), lymphomas (1% to 5%), gastrointestinal stromal tumors (2%), and carcinoid (1%).

Patients remain asymptomatic for years and consequently present late after the age of 65 years. Incidental or alarming signs and symptoms of gastric cancer are anorexia, nausea, vomiting, abdominal pain, dysphagia, postprandial fullness, chronic anemia, hematemesis, weight loss, and gastric outlet obstruction with succession splash [4].

## Case presentation

We report a case of a 63-year-old female with a medical history significant for a gastric bypass surgery (initial: 40 years ago, revision: 20 years ago), provoked deep venous thrombosis/pulmonary embolism 20 years ago, hypothyroidism, gastroesophageal reflux disease, and chronic low back pain, who presented to the hospital complaining of abdominal pain that started gradually 3 weeks ago and the associated symptoms including anorexia, nausea, and vomiting.

She underwent an abdominal computed tomography, which showed a marked distention of the gastric remnant with irregular thickening within the antro-pyloric region and the post-surgical changes of gastric bypass procedure showing a patulous segment of small bowel within the left mid-abdomen at the presumed jejunojejunal anastomosis.

**Figure 1:**
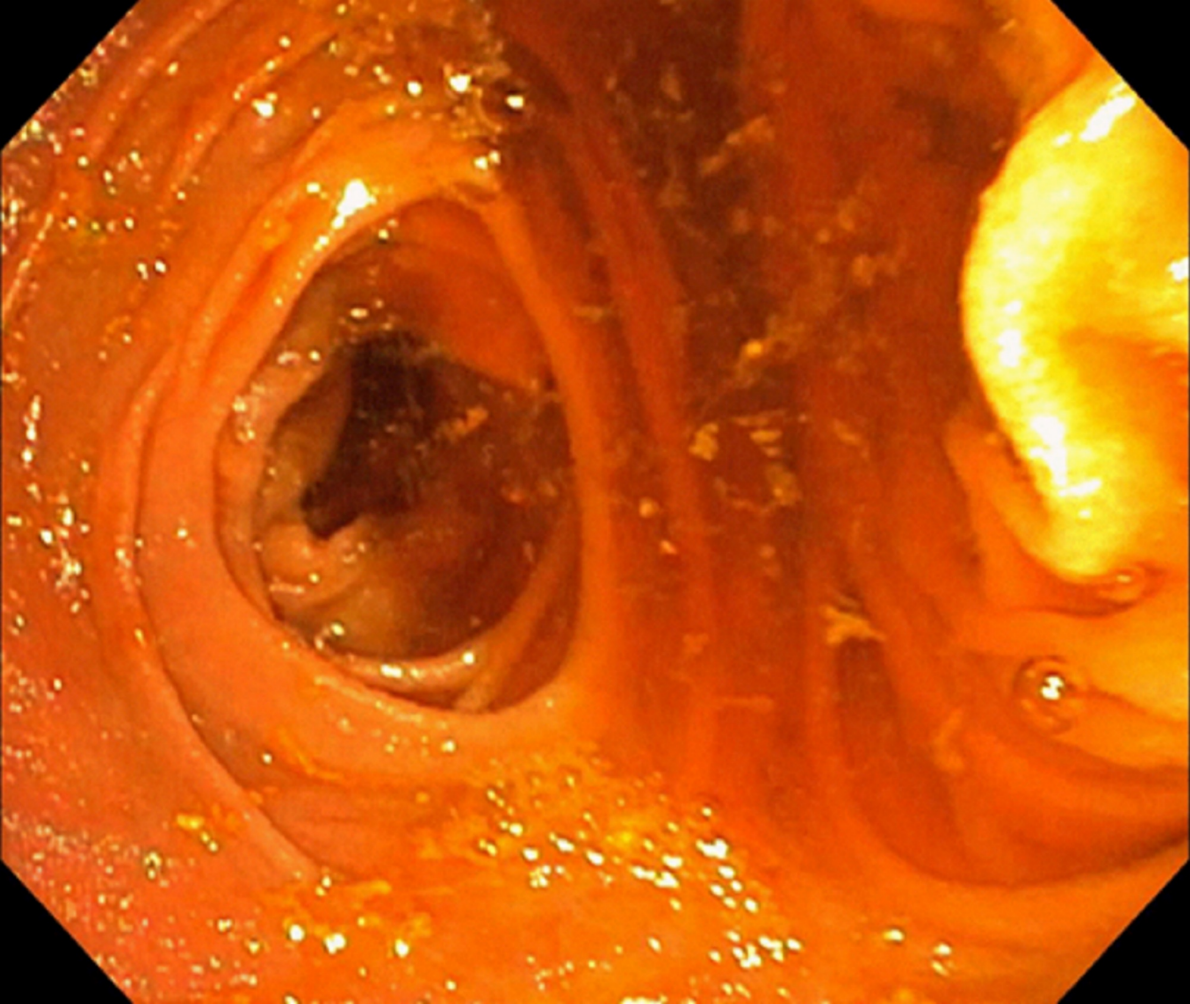
An enteroscopic view of the gastric lumen

**Figure 2:**
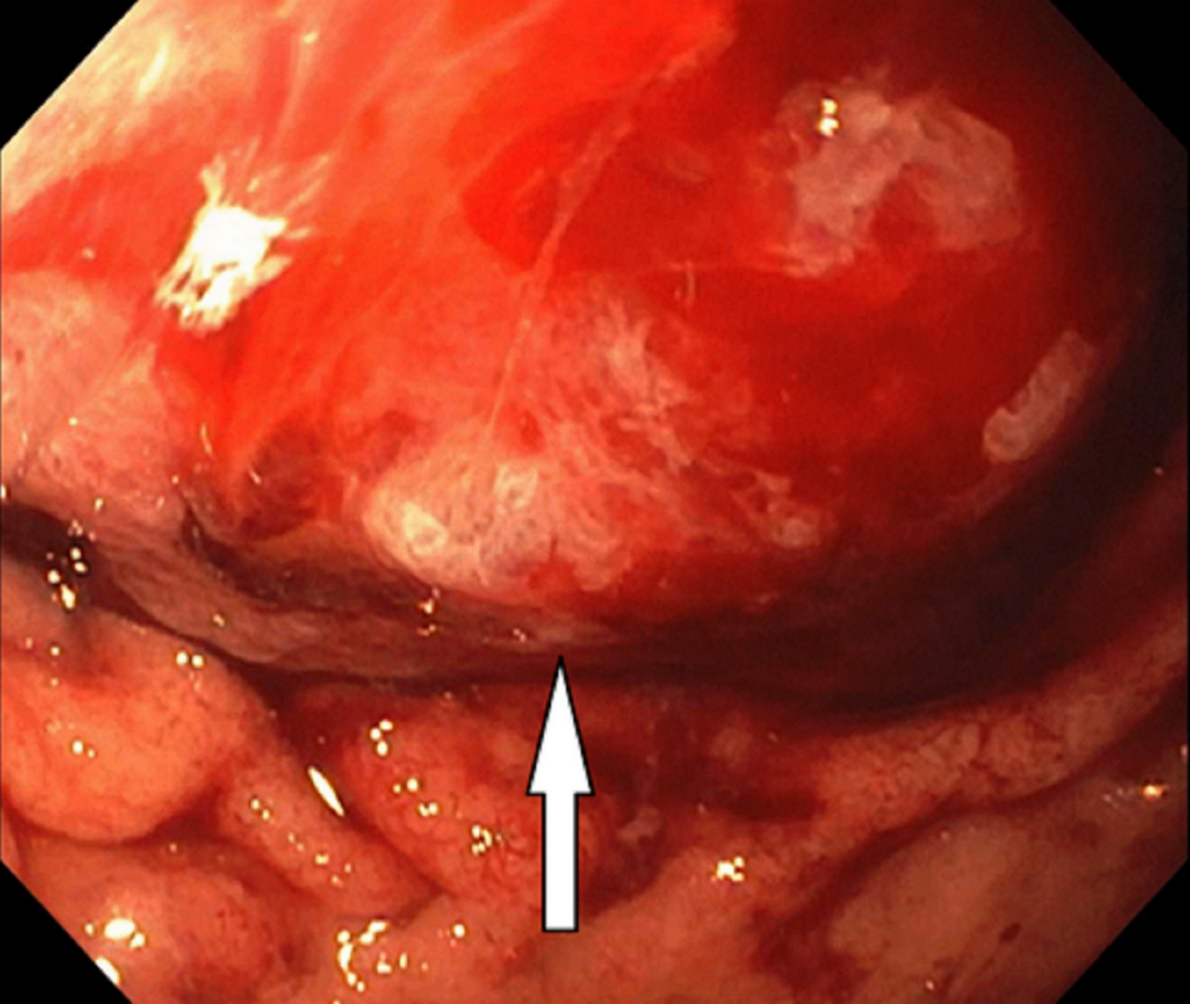
An irregular large frond-like villous mass at the pylorus

**Figure 3:**
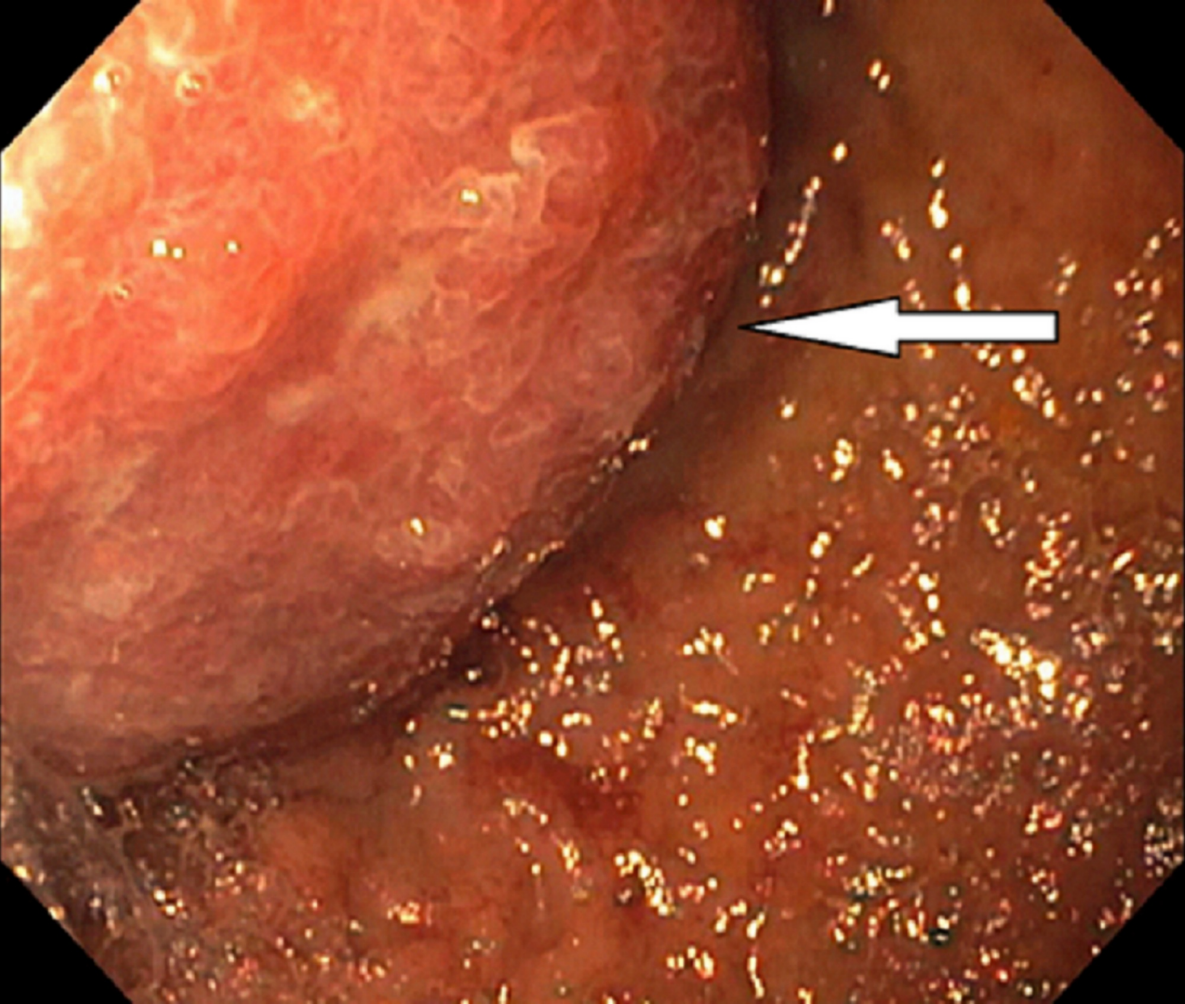
A mass protruding at the pylorus

Emergency esophagogastroduodenoscopy was done, which was unremarkable because of the inability of the scope to pass through the anastomosis following the bypass surgery. She underwent balloon-assisted retrograde enteroscopy, which showed a gastric bypass with a normal-sized pouch, an intact staple line, and gastro-jejunal anastomosis characterized by healthy appearing mucosa (Figure 1). The examined portion of jejunum was normal. A large frond-like villous mass was found at the pylorus with no evidence of bleeding (Figure 2). It was approached in a retrograde fashion and biopsied with cold forceps. The gastric remnant could not be evaluated, as the pyloric mass was obstructing the lumen (Figure 3).

Pathology work-up was expedited, and the results indicated adenocarcinoma in situ in the antro-pyloric region. Carcinoma in situ is an early stage of gastric cancer and falls under the category of Tis or stage 0; it is characterized by an intraepithelial tumor that does not invade the lamina propria as compared to the T1 stage tumor that invades the lamina propria, submucosa, and muscularis mucosa [5]. Her-2/neu testing was deferred because of the lack of definitive invasion.

## Discussion

Globally, stomach cancer has been ranked the fourth most common cancer [1]. In the past 70 years, the incidence of stomach cancer has decreased dramatically in the Western world, but it is still much more common in other parts of the world like East Asia and Europe [6]. This decrease in incidence is hypothesized to be due to improved dietary habits such as less consumption of salted and pickled foods or keeping food fresh using refrigerators.

The prevalence of obesity has led to an increase in the number of bariatric surgeries performed worldwide. The occurrence of gastric cancer after bariatric surgery is infrequent. The etiology has not been clearly decoded, but possible factors like chronic reflux of acid and stasis of food particles in the pouch can cause chronic mucosal irritation and consequent ischemic damage [7-8].

Risk factors for stomach cancer

Gastric cancer is interconnected to both inherited and environmental factors. Diet-related risk factors are foods rich in salts like cured fish and pickled or smoked foods high in nitrates [9]. Chronic inflammatory states affecting the gastrointestinal tract, such as atrophic gastritis or any radiation exposure, are some examples of the environmental factors. Smoking directly correlates with the increase in risk as well. *Helicobacter pylori*-induced chronic inflammation is still considered the strongest risk factor for gastric cancer. Previous gastric surgery also contributes as a risk factor, as it disturbs the normal pH of the stomach, leading to metaplasia and dysplasia in the luminal cells. Genetic factors also add to a portion of the stomach cancers. They include hereditary non-polyposis colorectal cancer (HNPCC), Peutz-Jeghers syndrome, familial adenomatous polyposis, and Li Fraumeni syndrome.

Stomach anatomy

The stomach comprises two parts: proximal and distal. The proximal part is further divided into cardia, fundus, and body, whereas the distal part is divided into antrum and pylorus. The smaller distal one-third part of the stomach is called antrum. Pylorus is the narrow connecting part between the stomach and the duodenum.

To investigate the cause of our patient’s symptoms, a detailed medical history was taken. A thorough physical examination was performed, and a basic lab work was done. Endoscopic biopsy is a highly sensitive and specific test and helps in early detection and treatment, hence changing the outcome of the disease. It not only helps in accurate visualization, but also provides the extent of the tumor, histopathology confirmation, typing, and staging. Single-balloon enteroscopy (SBE) was performed in our patient. Abnormal tissues seen on enteroscopic examination was biopsied, and the samples were sent to a pathologist for a histologic examination. A final pathologic diagnosis was made as adenocarcinoma in situ.

In 2001, double-balloon enteroscopy was introduced, which was then upgraded to SBE a few years later. The long, flexible endoscope (200 cm or 8 feet) goes deep into the small bowel and has an over-tube with a balloon at the tip. Repeated push and pull of the over-tube helps in reducing the loops of the small bowel, thus pleating the small bowel on the tube [10].

## Conclusions

Tortuous anatomy of the small bowel and its long length makes it challenging to examine at times. The Roux-en-Y is also an ultimate challenge to endoscopic procedures because it changes and remodels the gastrointestinal anatomy. The current advanced endoscopic techniques like single- or double-balloon enteroscopy have provided both therapeutic and diagnostic edge in the gastrointestinal imaging world. Moreover, many cases are identified in the advanced stage, despite a long nonspecific history of gastroesophageal reflux disease, vomiting, and epigastric pain, which leads to poor prognosis and high mortality. Radical gastrectomy with lymphadenectomy is the best approach that allows appropriate staging and offers long-term survival for patients with gastric adenocarcinoma.
